# Inhibition of IGF-1R Prevents Ionizing Radiation-Induced Primary Endothelial Cell Senescence

**DOI:** 10.1371/journal.pone.0078589

**Published:** 2013-10-24

**Authors:** Ronald Allan M. Panganiban, Regina M. Day

**Affiliations:** Department of Pharmacology, Uniformed Services University of the Health Sciences, Bethesda, Maryland, United States of America; Weizmann Institute of Science, Israel

## Abstract

Accelerated senescence is a primary response to cellular stresses including DNA damaging agents (e.g., ionizing radiation) and is widely believed to be caused by continuous proliferative signaling in the presence of cell cycle arrest. Studies of signal transduction pathways leading to accelerated senescence have revealed that inhibition of mammalian target of rapamycin (mTOR) by rapamycin rescues cells from senescence. However, the molecular mechanisms upstream of mTOR following ionizing radiation (IR) are not well defined. We investigated signal transduction leading to IR-induced accelerated senescence in human pulmonary artery endothelial cells (HPAEC). Exposure of HPAEC to X-rays (10 Gy, 2.4 Gy/min) upregulated senescence markers including p53, p21/waf1, and senescence-associated beta galactosidase (SA-β-gal). Ly294002 (a phosphatidylinositol-3-kinase [PI3K] inhibitor) or rapamycin (an mTOR inhibitor) blocked the induction of cellular senescence markers suggesting roles for PI3K and mTOR. Pathway-directed microarrays revealed increased transcription of insulin-like growth factor I (IGF-1), a modulator of cell growth and proliferation upstream of mTOR. qRT-PCR confirmed that both IGF-1 and IGF-2 mRNA were increased in response to X-rays, and ELISA showed increased secretion of IGF-1 protein into the medium of irradiated HPAEC. Consistent with upregulation of these ligands, we found that X-ray exposure led to hyperphosphorylation of IGF-1R, the receptor for IGF-1 and -2. Treatment with AG1024, an IGF-1R inhibitor, suppressed IR-induced upregulation of p53, p21/waf1, and SA-β-gal. Together these findings suggest that IGF-1R is a key regulator of IR-induced accelerated senescence in a pathway that requires intact mTOR activity upstream of both p53 and p21/waf1.

## Introduction

Accelerated senescence is a well-recognized cellular response to environmental stresses that damage biological molecules especially DNA. It is characterized by loss of replicative capacity, abnormal gene expression of cell cycle regulators, altered responsiveness to apoptotic stimuli, alterations in cellular morphology, induction of senescence-associated secretory proteins, and increased senescence-associated beta-galactosidase (SA-β-gal) activity [1]. Accumulating body of evidence implicates a role for cellular senescence in aging, suppression of tumorigenesis, and overall tissue and organ dysfunction possibly through depletion of functional cells required for organ homeostasis and through induction of inflammation associated with the secretory phenotype [1-3].

Recent studies suggest that accelerated senescence occurs as a result of proliferative signaling in the presence of a cell cycle checkpoint blockade, often p21/waf1 [4,5]. The mammalian target of rapamycin (mTOR), a cytoplasmic kinase that is widely regarded as a central integration point for a number of cell signaling pathways including cell proliferation and homeostasis [6], has been identified as a central molecular target for the inhibition of replicative senescence as well as stress-induced cellular senescence [4,5,7,8]. Treatment with rapamycin, an mTOR inhibitor, prevents accelerated senescence in cells exposed to DNA-damaging agents [5,9]. Similarly, paradoxically, both mitogen activated protein kinase (MAPK) p42/p44 and phosphatidylinositol-3-kinase (PI3K)/Akt signaling pathways which play roles in cell survival and proliferation have also been shown to positively regulate the development of senescence [10-12]. Akt deficiency causes resistance to replicative- and stress-induced premature senescence while its activation induces premature senescence via increased production of reactive oxygen species (ROS) [10]. On the other hand, MAPK p42/p44 mediates thrombopoietin-induced senescence during megakaryocytic maturation [13]. Both signaling pathways appear to require increased expression of the cell cycle checkpoint protein p21/waf1 for the induction of cellular senescence [13,14].

Investigation into receptor signaling pathways that contribute to aging-associated cellular senescence has revealed the possible involvement of the insulin like growth factor-1 receptor (IGF-1R) [15,16]. IGF-1R is a single transmembrane tyrosine kinase receptor whose ligands include IGF-1 and IGF-2 [17]. The activation of IGF-1R involves autophosphorylation of its intracellular domain, followed by recruitment of docking intermediates including the insulin-receptor substrate-1 (IRS-1), which in many cell types leads to activation of PI3K/Akt, MAPK, and mTOR [18-21]. As a growth factor receptor, IGF-1R is known to play a role in cell growth and proliferation under normal conditions and is widely expressed in most transformed cells, conferring pro-survival properties upon stress application [21-24]. In agreement with the hypothesis that IGF-1R acts as a mediator of cell survival and proliferation, a number of studies showed a positive correlation between activation of IGF-1R and radiation resistance in some cells [25-28]. However, most of these studies were focused only on the contribution of apoptosis and it is likely that IGF-1R operate on other modes of radiation-induced cellular response depending on the cellular context. Moreover, the anti-apoptotic activity of IGF-1R appears to be dispensable for the induction of radiation resistance in a variety of tumor cells suggesting the possibility of an unidentified mechanism [29]. Although the IGF-1,-2/IGF-1R signaling axis is known to promote cell proliferation and survival under most circumstances, IGF-1R was recently implicated in several models of senescence. IGF-1R expression levels increased during the development of *in vitro* replicative senescence in primary cortical neurons [30]. UVB-induced premature senescence was found to require functional IGF-1R in human keratinocytes [15]. IGF-1 also enhanced senescence in primary cell cultures via a mechanism that involved increased reactive ROS generation leading to induction of the p53/p21 pathway [31]. In mouse embryonic fibroblasts, treatment with IGF-1 inhibited the DNA deacetylase activity of Sirtuin 1 (SIRT1) and promoted stability of p53, ultimately leading to induction of senescence [32]. 

In our previous studies, we determined that accelerated senescence is the primary response of normal PAEC to X-ray exposure [33]. We now provide evidence for the involvement of IGF-1R in the development of IR-induced accelerated senescent phenotype in primary lung endothelial cells. Our results suggest that IGF-1R signaling is required for X-ray-induced accelerated senescence in endothelial cells. 

## Materials and Methods

### Cell Culture and Reagents

Human pulmonary artery endothelial cells (HPAEC) were obtained from Cell Applications, Inc. (San Diego, CA) and cultured in EBM-2 basal medium containing supplements and growth factors as indicated in the manufacturer’s protocol (Lonza, Walkersville, MD). Cells were maintained at 37°C in a humidified atmosphere containing 5% CO_2_. Subconfluent HPAEC at passages 4-8 were used for all experiments. The following chemical inhibitors and their final concentrations were used: AG1024 (5 μM, Calbiochem, EMD Millipore, Billerica, MA), Ly294002 (20 μM, Calbiochem), rapamycin (500 nM, Calbiochem), U0126 (10 μM, Cell Signaling Technology, Danvers, MA). Each inhibitor was dissolved in DMSO and added to cell cultures so that the final concentration of the solvent did not exceed 0.1%. N-acetyl cysteine (NAC, 20 mM) Calbiochem) was dissolved in distilled H_2_0.

### Cell Irradiation

HPAEC were either irradiated or sham-irradiated at subconfluence (70-90%). Irradiations were conducted using RS2000 Biological Irradiator (Rad Source Technologies, Alpharetta, GA) with 0.3 mm Cu shielding at a dose rate of 2.4 Gy/min (160kV, 25mA) at room temperature. 

### Western blotting

Whole cell extracts were prepared in RIPA buffer (50 mM Tris HCl pH 8, 150 mM NaCl, 1% NP-40, 0.5% sodium deoxycholate, and 0.1% SDS) supplemented with protease inhibitors (Sigma-Aldrich, St. Louis, Montana), 1 mM PMSF, 2 mM Na_3_VO_4_ and/or Halt phosphatase inhibitors (Thermo Scientific, Rockford, IL) or SDS-laemmli buffer (Bio-Rad, Hercules, CA) containing 50 mM DTT. Samples were vortexed, incubated for 10 min at 4°C, and subjected to sonication (Heat Systems-Ultrasonics Inc., Plainview, NY) for 5 sec at 4°C. Samples were then centrifuged at 14,000 x *g* for 10 min and supernatant was collected. 

 Protein concentrations from whole cell lysates were determined using Protein® BCA Protein Assay Kit (Thermo Scientific, Rockford, IL). Equal amounts of proteins were separated in SDS-PAGE and transferred to PVDF membranes (Thermo Scientific, Rockford, IL). For western blotting, the following antibodies were used: anti-phospho-IGF1R (Tyr1165/1166), total IGF-1R, anti-p53, anti-p21/waf1, and anti-β-actin (Santa Cruz Biotechnology, Inc., Santa Cruz, CA); anti-phospho-S6 (Ser 235/236), anti-phospho-Akt (Ser 473) and total Akt (Cell Signaling Technology, Danvers, MA). Proteins were detected with horseradish peroxidase-linked secondary antibodies and SuperSignal West Pico Chemiluminescent Substrate (Pierce, Rockford, IL). 

### Quantitative real-time reverse transcription polymerase chain reaction (qPCR)

Total RNA was isolated from cultured cells using the RNeasy Kit (Qiagen, Valencia, CA). RNA was quantified spectroscopically (ND-1000 Spectrophotometer, NanoDrop, Wilmington, DE). RNA (400 ng) was subjected to reverse transcription in a total volume of 20 μl. Following dilution 5-fold with water, 2 μl of cDNA was used for 20 μl qPCR reaction. PCR was performed in duplicates using 6 μM of each primer and 10 μl of SybrGreen® PCR master mix (Applied Biosystems, Foster City, CA). The following primers were used for detection of IGF-1: forward 5’- TGC CCA AGA CCC AGA AGT -3’ and reverse 5’- CTC CTG TCC CCT CCT TCT GTT - 3’ and IGF-2: forward 5’- ACA CCC TCC AGT TCG TCT GT- 3’and 5’- and GAAACAGCACTCCTCAACGA-3’. As an internal control, mRNA levels of GAPDH were determined using primers: forward 5’-GAA GCT CGT CAT CAA TGG AAA-3’ and reverse 5’-CCA CTT GAT GTT GGC AGG AT-3’. For quantification, the comparative threshold cycle (Ct) method was used to assess relative changes in mRNA levels between the untreated (control) and the irradiated samples. 

### Senescence-associated β-galactosidase (SA-β-gal) Assay

SA-β-gal assay was performed according to established protocols with minor modifications [34,35]. To avoid density-dependent false positive expression of SA-β-gal, HPAEC were seeded at a density of 1.0-2.0 x 10^4^ on 12-well or 6-well dishes and allowed to reach 50-70% confluence prior to treatment. At indicated time-points postirradiation, cells were washed twice in ice cold PBS, fixed in 2% formaldehyde/ 0.2% glutaraldehyde in PBS for 5 min at room temperature and washed again twice in ice-cold PBS. 1.5-2 ml of freshly prepared X-gal staining solution (1 mg/ml of 5-bromo-4-chloro-3-indolyl β-D-galactoside in 40 mM citric acid/sodium phosphate, pH 6.0 (made by mixing 36.85 parts 0.1 M citric acid solution with 63.15 parts 0.2 M sodium phosphate solution and then verifying pH to be 6.0, 0.1M citric acid was added to adjust pH when necessary), 5 mM potassium ferrocyanide, 5 mM potassium ferricyanide, 150 mM NaCl, 2 mM MgCl_2_) was then added to the culture dishes and the dishes were incubated for 12-20 h at 37°C (without CO_2_). After incubation, cells were washed twice in ice-cold PBS and once in methanol and allowed to dry. Cells were examined for perinuclear blue staining indicative of SA-β-gal activity in at least 3 random fields. 

### Statistics

Means ± standard deviations (SD) or error of the mean (SEM) were calculated, and statistically significant differences between two groups were determined by the Student's *t* test. p <0.05 was considered statistically significant.

## Results

### HPAEC undergo accelerated senescence post-irradiation

**Figure 1:**
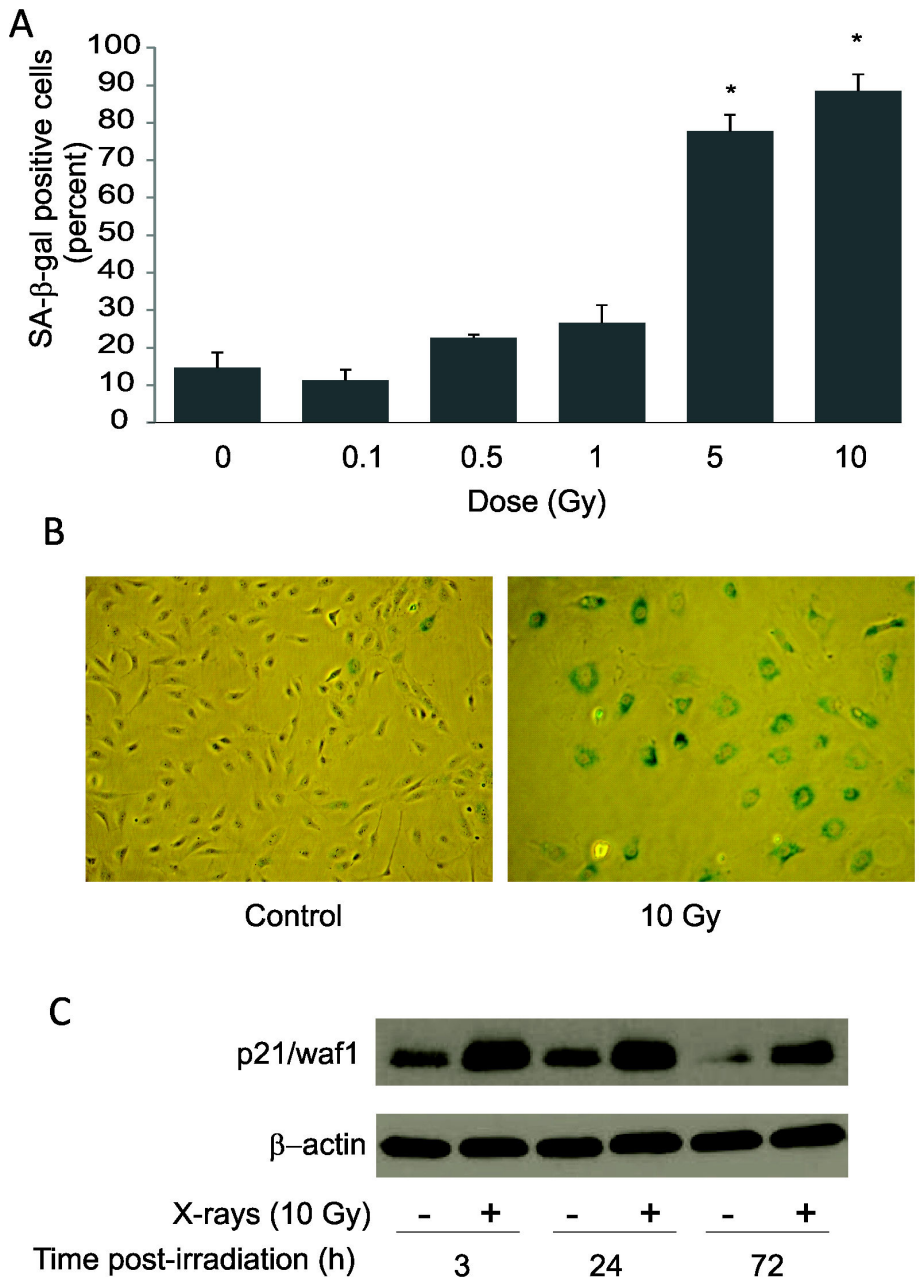
X-rays induce accelerated senescence in HPAEC. Subconfluent HPAEC were exposed to indicated doses of X-rays and then incubated until time of assay. (A) Dose-response effect of increasing X-ray doses in HPAEC. At 4 days post-irradation, irradiated and sham-irradiated HPAEC were assayed for SA-β-gal activity. Percentage of SA-β-gal positive cells was counted in at least 3 random fields. Graph represents means ± SEM, n = 3. * indicates statistical significance from controls, p<0.05. (B) SA-β-gal staining sham-irradiated and irradiated (10 Gy) (C) Time-course of p21/waf1 upregulation. Western blotting for p21/waf1 expression at indicated time-points post-irradiation.

We previously determined in bovine PAEC that accelerated senescence is the primary cellular response to exposure to 10 Gy X-rays with very limited apoptosis and no detectable necrosis [33]. Two salient features of senescence were identified in X-ray-induced senescence in bovine PAEC, upregulation of p21/waf1 and increased SA-β-gal activity. The cyclin-dependent kinase inhibitor, p21/waf1, is a cell cycle checkpoint protein and contributes to cell cycle arrest, a necessary component of cellular senescence. SA-β-gal is a widely used marker for cellular senescence that was first described by Dimri et al. [34] and has routinely been used over the years to detect senescent cells *in vitro* and *in vivo* [36]. We investigated the effects of X-ray exposure on the development of senescent phenotype in human PAEC (HPAEC). Our data indicated that HPAEC underwent cellular senescence upon exposure to 10 Gy X-rays as determined by increased SA-β-gal activity, detected cytochemically as blue perinuclear staining at 4 days post-irradiation. (Figure 1A,B). X-ray-exposed HPAEC also exhibited changes in cell morphology, displaying unusually large cells and flattened cytoplasmic appearance compared to sham-irradiated controls (Figure 1B). We also observed the upregulation of p21/waf1 within 3h post-irradiation, consistent with inhibition of the cell cycle in advance of senescence (Figure 1C). These data are consistent with our findings using bovine derived PAEC [33]. 

### X-rays activate the IGF-1R signaling cascade

**Figure 2:**
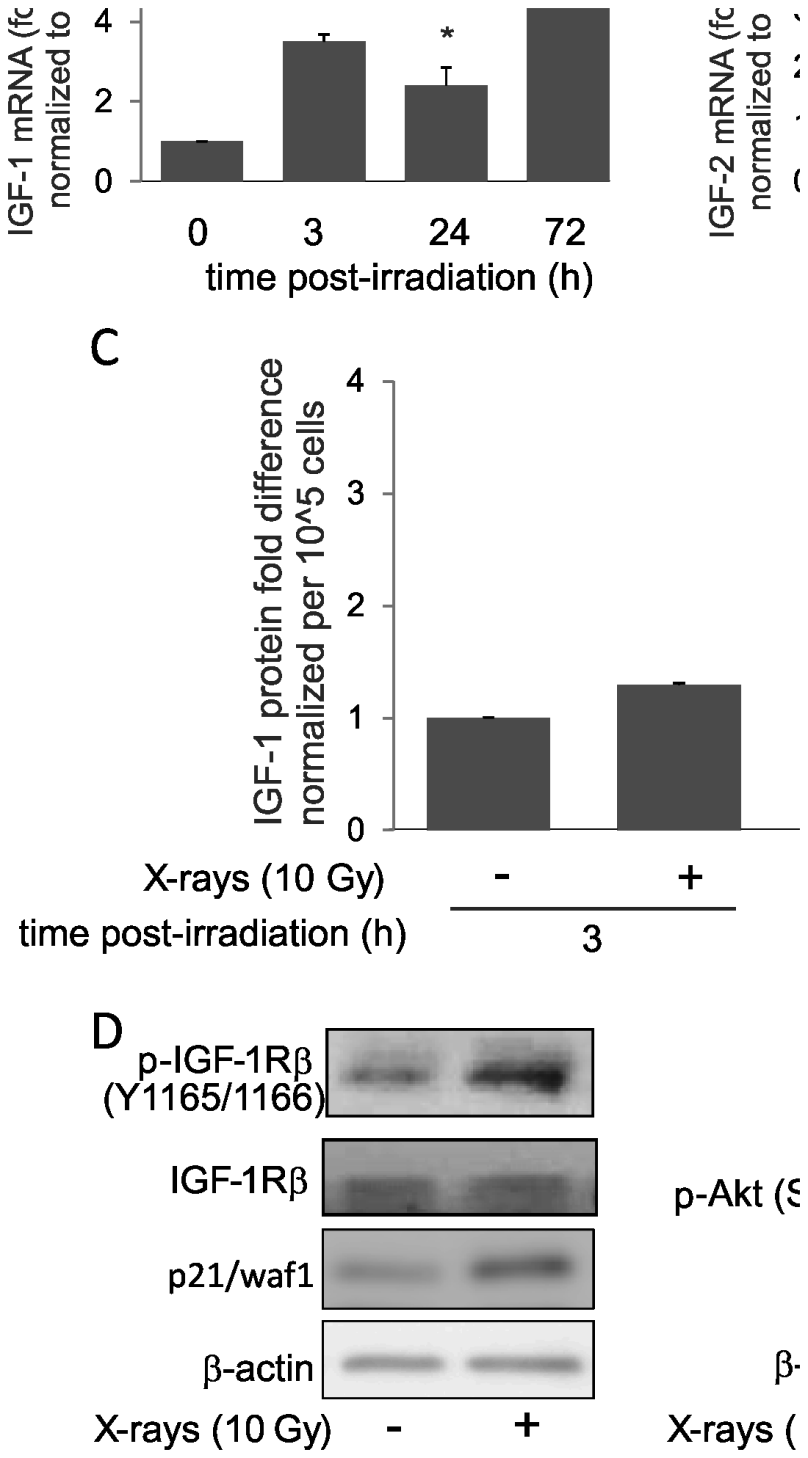
Induction of IGF-1R signaling in irradiated HPAEC. (A and B) IGF-1 and IGF-2 mRNA levels in irradiated HPAEC were assessed by qPCR at indicated time-points post-irradiation. The mRNA levels were normalized to GAPDH. Graph represents means ± SD, n = 3. * indicates statistical significance from controls, p < 0.05. (C) IGF-1 levels in secreted medium were measured by ELISA at indicated time-points post-irrradiation. Graph represents means ± SD, n = 3. * indicates statistical significance from controls, p < 0.05. (D) Western blotting for expressions of proteins involved in IGF-1R hyperphosphorylation (left panel) and Akt hyperphosphorylation (Ser 473, right panel) at 3 hours post-irradiation.

Exposure to X-rays causes alterations in global gene expression. As we had previously determined that the primary response causing loss of cell viability in PAEC is accelerated senescence, we used a pathway-focused senescence array to examine the changes in mRNA level over time after 10 Gy exposure in HPAEC. Among the genes observed to be altered in expression, IGF-1 mRNA level was increased ~15 fold within 72 hours post-irradiation (Table S1). We confirmed IGF-1 mRNA findings using qRT-PCR (~2 fold at 24 hours post-irradiation and ~6 fold at 72 hours post-irradiation, p<0.05, Figure 2A). Similarly, IGF-2 mRNA was significantly increased post-irradiation (~ 1.7 fold at 24 hours and ~1.5 fold at 72 hours, Figure 2B). IGF-1 can act in paracrine and/or autocrine manner [37] and in cultured cells it is secreted in the medium once produced [38,39]. ELISA assays indicated that the IGF-1 level in the medium was increased 72 hour post-irradiation (~3.2 fold, p<0.05, Figure 2C). These increases in the secretion of IGF-1 and in the gene expressions of both IGF-1 and IGF-2, two known activating ligands of IGF-1R, led us to examine the activation of its receptor IGF-1R. Phosphorylation at Y1165 and Y1166 is essential for IGF-1R kinase activation [40,41]. Phosphorylation at Ser 473 is required for full activation of Akt in a PI3K-dependent manner [42]. We detected increased IGF-1R phosphorylation within 3 hours post-irradiation along with Akt hyperphosphorylation (Figure 2D). Interestingly, the increase in phosphorylation of IGF-1R and Akt occurred concurrently with p21/waf1 upregulation.

### AG1024 blocks radiation-induced accelerated senescence

**Figure 3:**
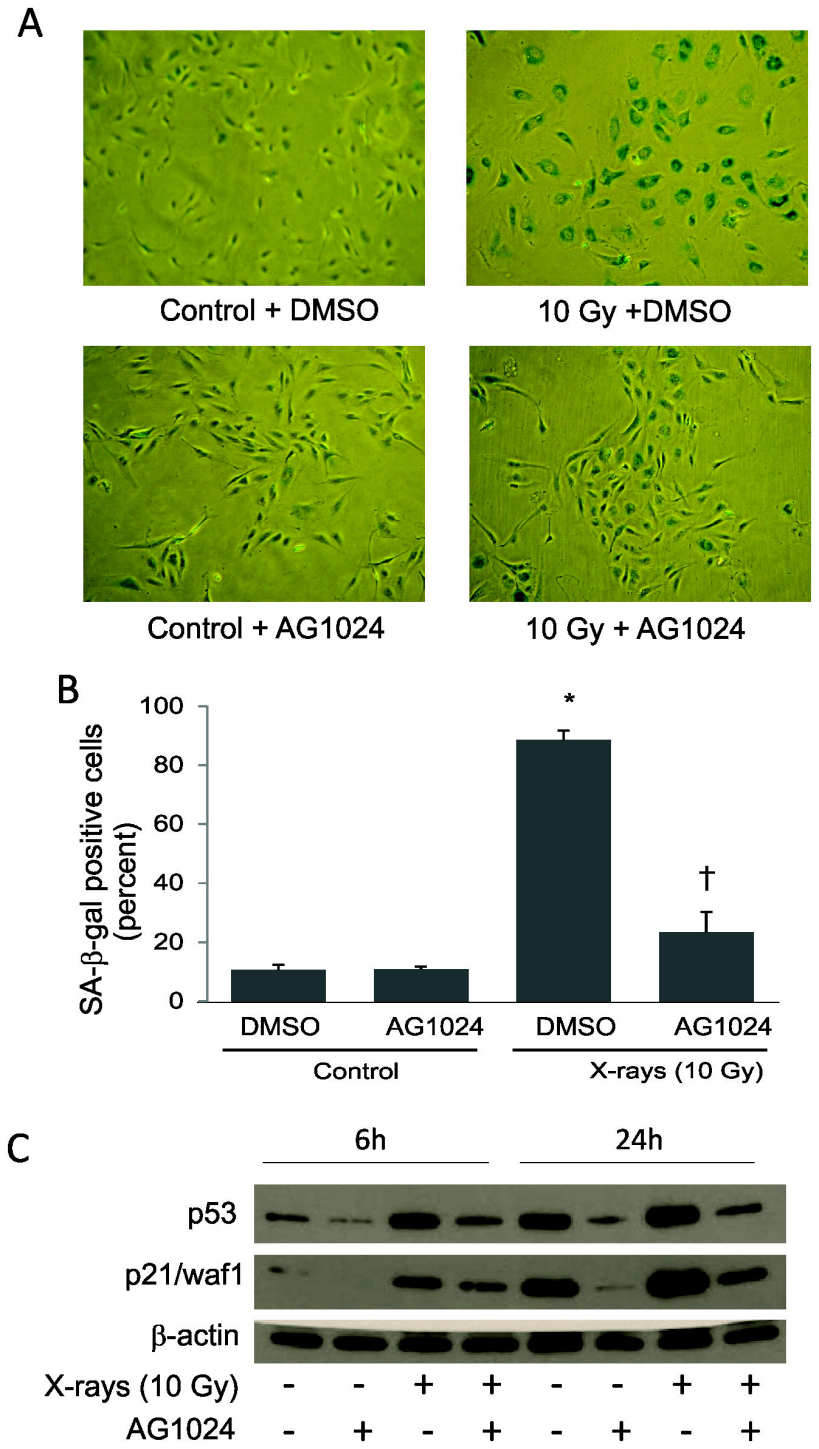
Attenuation of radiation-induced accelerated senescence by AG1024. Subconfluent HPAEC were pretreated with 5 μM AG1024 or vehicle (DMSO) for 30 minutes, exposed to 10 Gy X-rays and then incubated until time of assay. (A) Representaive pictures of SA-β-gal staining at 4 days post-irradiation (B) Percentage of SA-β-gal positive cells was counted in at least 3 random fields. Graph represents means ± SEM, n = 6. * indicates statistical significance from controls, p<0.05. (C) Representative western blotting for p53 and p21/waf1 expression at indicated time-points post-irradiation in the presence of AG1024 or DMSO.

As X-rays induced IGF-1R phosphorylation, IGF-1 and IGF-2 upregulation as well as accelerated senescence in HPAEC, we hypothesized that IGF-1R activation may function in the development of accelerated senescence post-irradiation. To test this hypothesis, we exposed HPAEC to 10 Gy X-rays in the presence of AG1024 (an IGF-1R inhibitor) and examined cellular senescence. Inhibition of IGF-1R prevented the change in cellular volume observed within 96 h of radiation exposure, and cells maintained a more normal morphology (Figure 3A). Blocking IGF-1R activation significantly reduced cellular senescence as shown by suppression of radiation-induced p53 and p21/waf1, and by radiation-induced SA-β-gal activity assay (Figure 3C and 3B). 

### Inhibition of mTOR and PI3K, but not p42/p44 MAPK, blocks radiation-induced accelerated senescence

**Figure 4:**
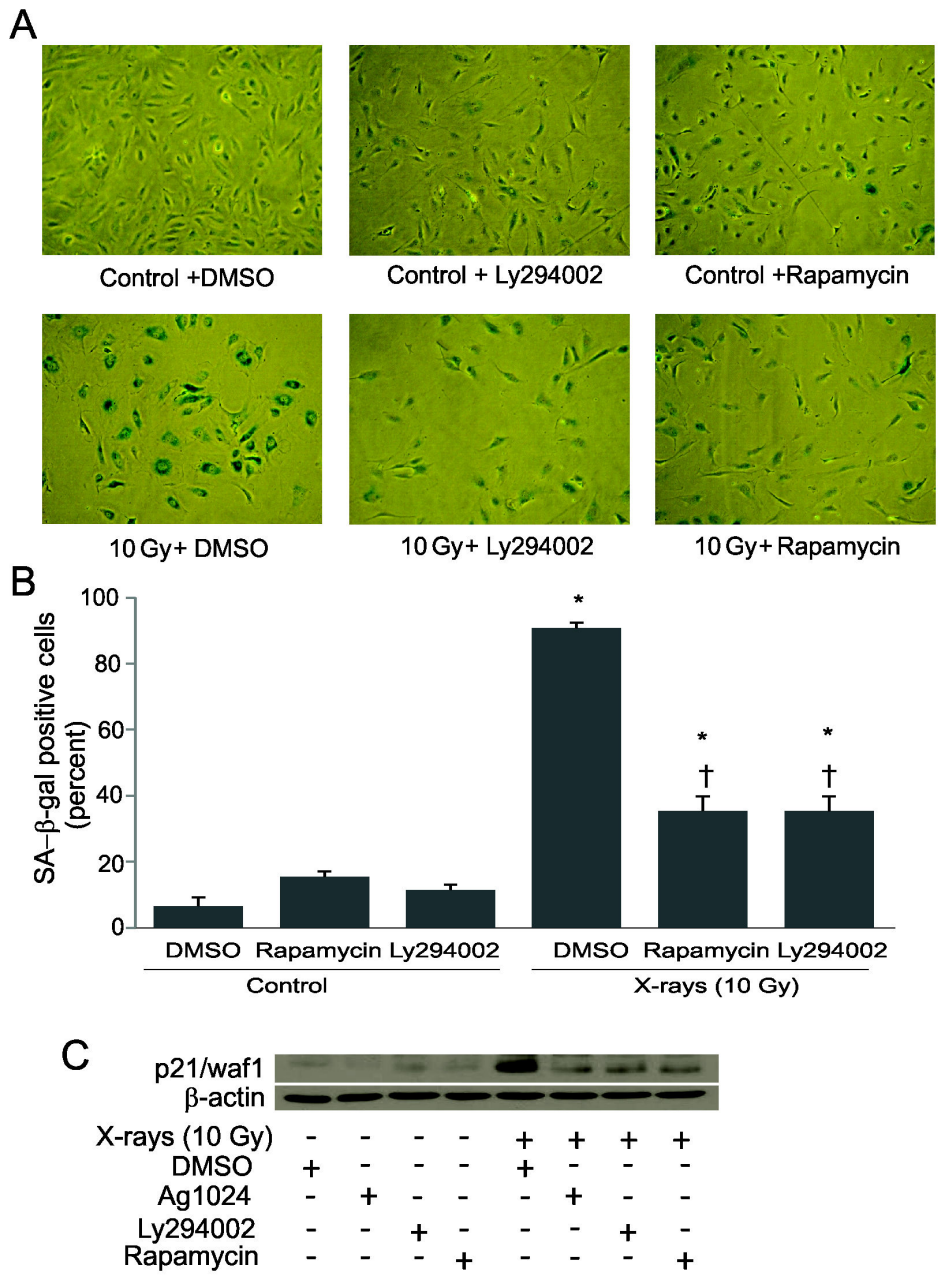
Attenuation of radiation-induced accelerated senescence by rapamycin and Ly294002. Subconfluent HPAEC were pretreated with 500 nM rapamycin, 20 μM Ly294002, or vehicle (DMSO) for 30 minutes, exposed to 10 Gy X-rays and then incubated until time of assay. (A) Representative pictures of SA-β-gal staining at 4 days post-irradiation (B) Percentage of SA-β-gal positive cells was counted in at least 3 random fields. Graph represents means ± SEM, n = 6. * indicates statistical significance from controls, p < 0.05. (C) Representative western blotting for p21/waf1 expression at indicated time-points post-irradiation in the presence of AG1024, rapamycin, Ly294002 or DMSO.

Recent studies demonstrated that mTOR is required for stress-induced senescence. Others have demonstrated that treatment with the mTOR inhibitor rapamycin prevented the increase in SA-β-gal activity in cells exposed to DNA-damaging agents [5,43,44] and protected mice from radiation-induced mucositis *in vivo* [45]. Furthermore, IGF-1R signaling activates PI3K/Akt pathways which leads to mTOR activation [46,47]. We tested whether inhibition of mTOR or PI3K would attenuate IR-induced accelerated senescence in irradiated HPAEC. The mTOR inhibitor rapapmycin and the PI3K inhibitor LY294002 both resulted in the maintenance of normal cellular morphology following 10 Gy X-ray exposure (Figure 4A). Inhibition of PI3K and mTOR also attenuated radiation-induced SA-β-galactosidase activation (Figure 4A, B) and attenuated radiation-induced p21/waf1 expression (Figure 4C). Interestingly, treatment with U0126, a MAPK inhibitor, did not rescue cells from undergoing accelerated senescence (Figure S1).

**Figure 5:**
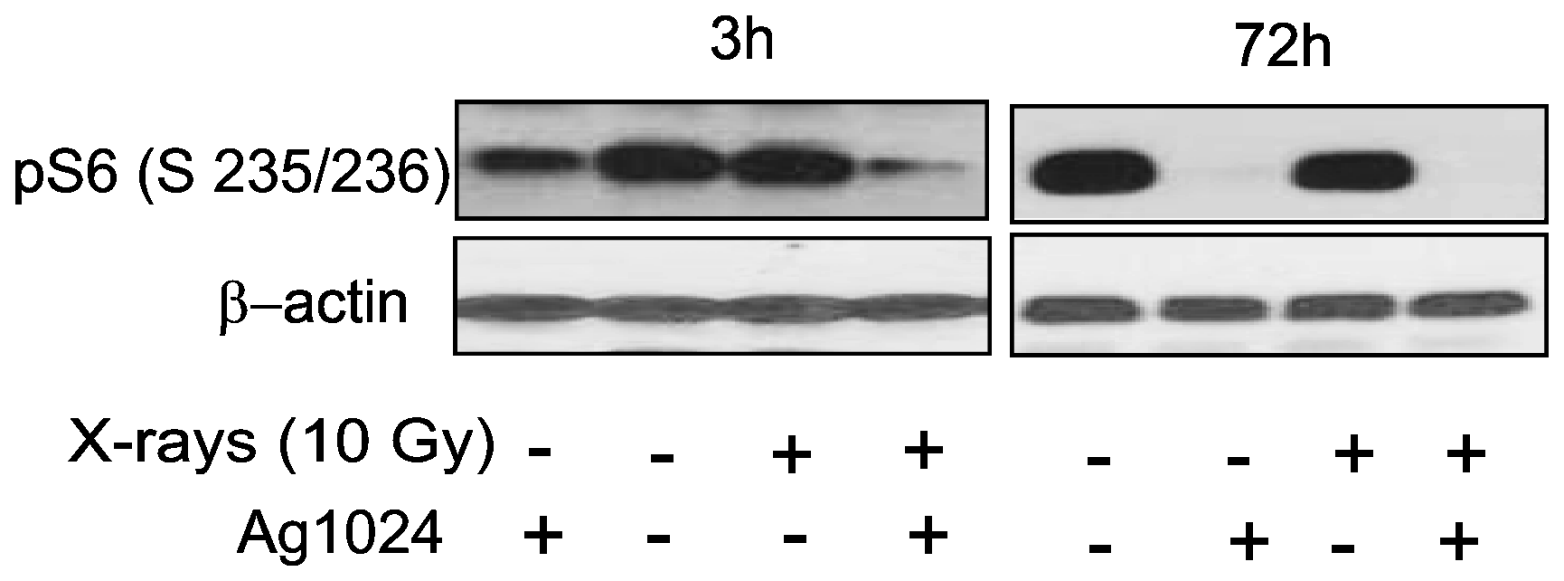
IR-induced IGF-1R-mediated accelerated senescence requires intact mTOR. Subconfluent HPAEC were treated with 5 μM AG1024 or DMSO for 30 minutes and subjected to 10 Gy X-rays. At 3 and 72 hours post-irradiation, whole cell lysates were prepared and western blotting for phospho-S6 (Ser 235/236) was performed.

We investigated the dependence of Akt activation by X-ray irradiation on IGF-1R signaling. We found that the inhibition of IGF-1R by AG1024 had no effect on radiation-induced phosphorylation of Akt S473 (unpublished results). To determine whether IGF-1R signaling is upstream of mTOR activation following radiation exposure, we examined the effect of AG1024 on phosphorylation of S6 ribosomal protein (pS6), a known target of S6 kinase which is downstream of activated mTOR [48]. AG1024 treatment significantly reduced the phosphorylation of S6 ribosomal protein (Ser 235/236, Figure 5) suggesting that intact mTOR activity is required for radiation-induced IGF-1R-mediated accelerated senescence.

### Radiation-induced reactive oxygen species are required for early IGF-1R phosphorylation

**Figure 6:**
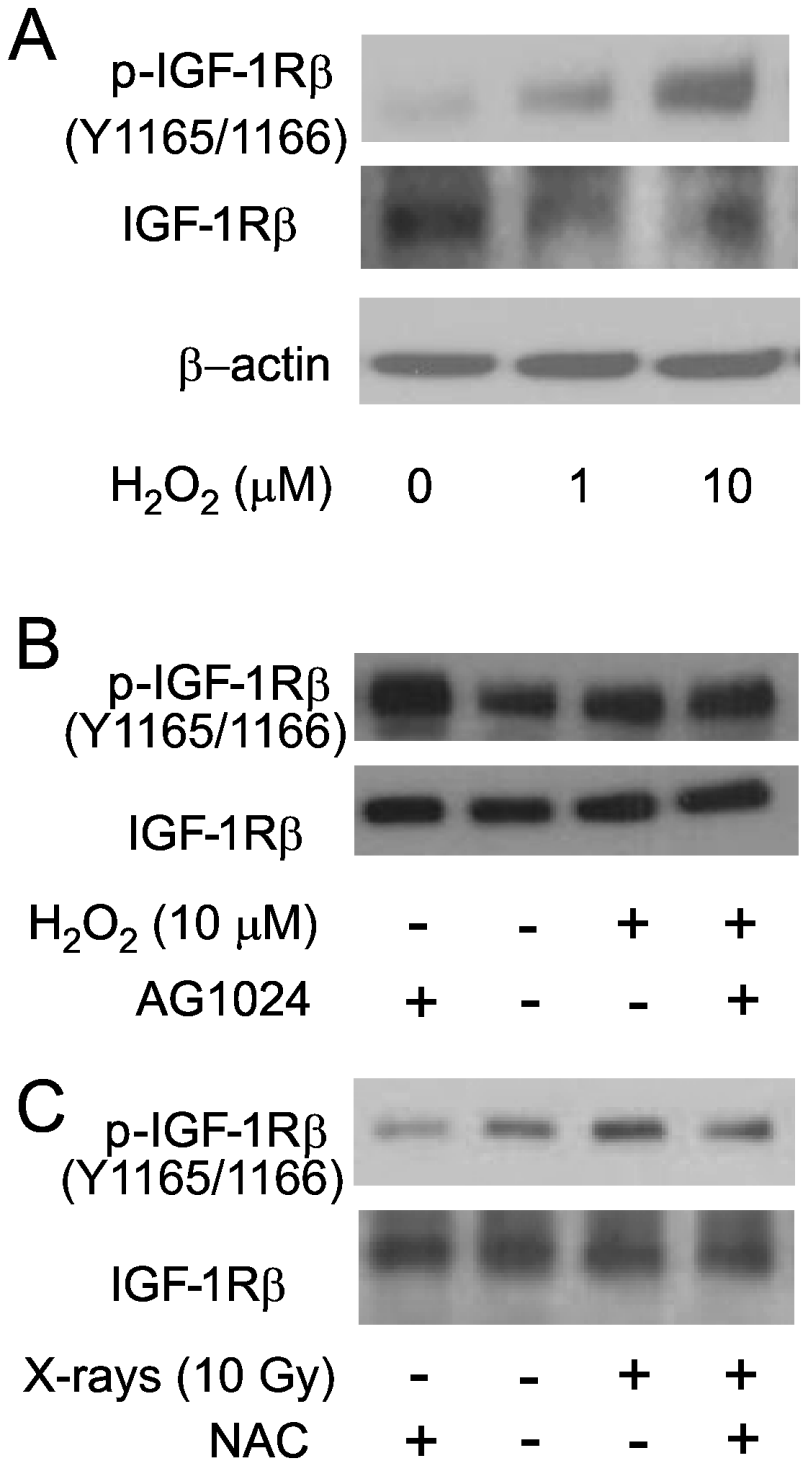
H_2_O_2_ induces phosphorylation of IGF-1R. (A) Subconfluent HPAEC were treated with 1 μM and 10 μM H_2_O_2_ and incubated in a CO_2_ incubator. After 20 minutes, whole cell lysates were prepared and western blotting was performed to determine the phosphorylation status of IGF-1R. (B) HPAEC were treated with 5 μM AG1024 for 30 minutes and then with 10 μM H_2_O_2_ for 20 minutes. Western blotting was then performed to determine phosphorylation status of IGF-1R. (C) HPAEC were treated with 20 mM NAC and subjected to 10 Gy X-rays. Western blotting was then performed 3 hours post-irradiation to determine phosphorylation status of IGF-1R.

Our data indicated that IGF-1R phosphorylation could be detected at time points that preceded the increased production of IGF ligands. We therefore investigated alternative mechanism of early IGF-1R activation. Exposure to IR is known to cause accumulation of ROS via radiolysis of intracellular H_2_O and through subsequent production of intracellular ROS [49,50]. ROS have also been shown to cause phosphorylation of cellular receptors, including IGF-1R in vascular smooth muscle cells [51,52]. To determine whether ROS alone would be sufficient for the induction of IGF-1R phosphorylation, we treated HPAEC with H_2_O_2_ (1 μM and 10 μM) for 20 minutes. As shown in Figure 6A, H_2_O_2_ alone was sufficient to induce IGF-1R phosphorylation. We then determined whether this ROS-induced phosphorylation is ligand-dependent by treating the cells with H_2_O_2_ in the presence of AG1024 and blotting for phospho-IGF-1R. As shown in Figure 6B, ROS-induced IGF-1R phosphorylation was not inhibited by AG1024. Finally, to determine whether IR-induced IGF-1R phosphorylation is mediated by ROS, we treated HPAEC with 20 mM N-acetyl-L-cysteine (NAC) for 1 hour and exposed the cells to 10 Gy X-rays. Western blotting shows that IGF-1R phosphorylation was attenuated in the presence of NAC (Figure 6C).

### Treatment with AG1024 or increasing the concentration of IGF-1 has no effect on radiation-induced caspase-3 activation

**Figure 7:**
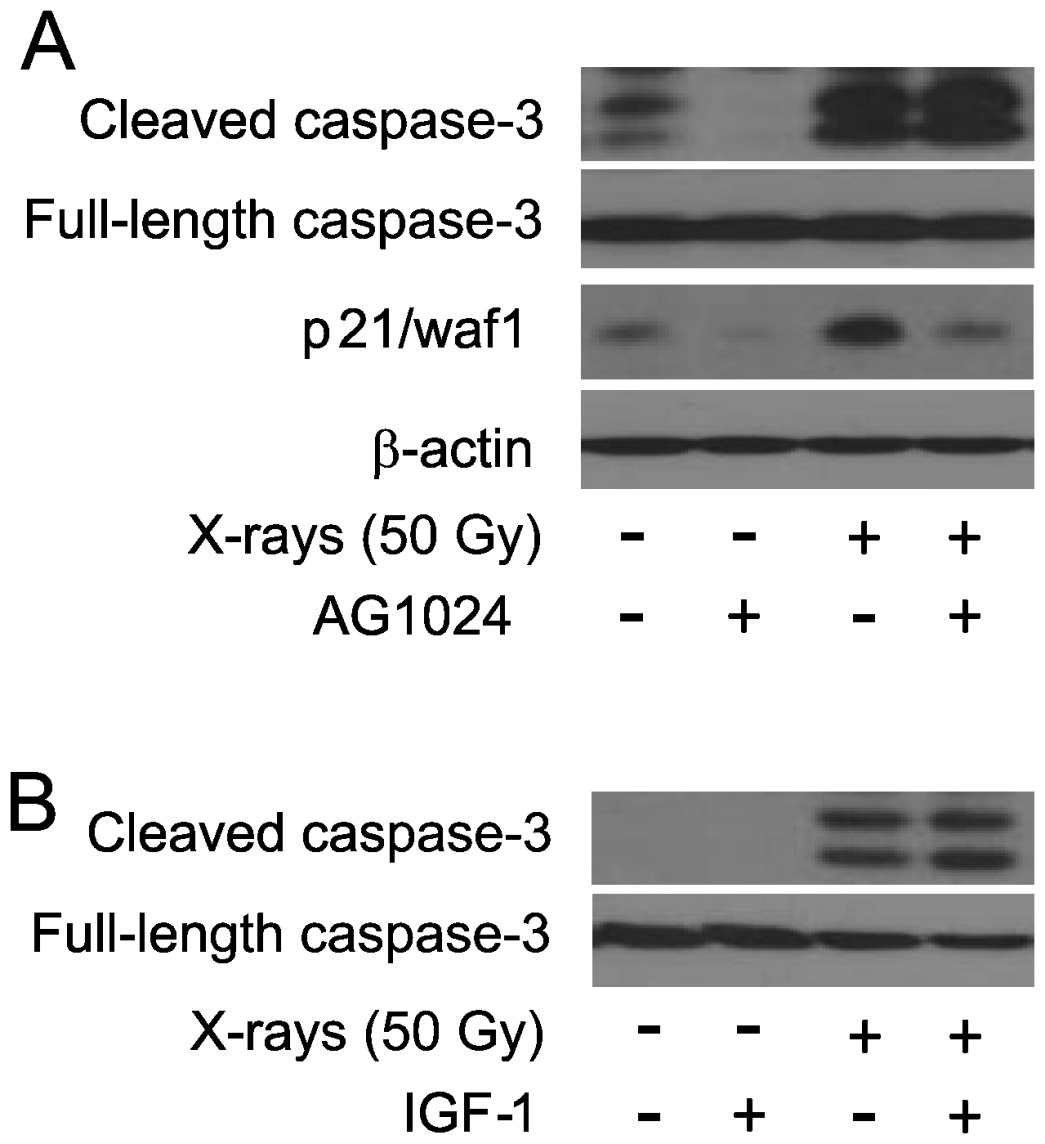
Treatment with AG1024 or addition of IGF-1 does not regulate IR-induced apoptosis. (A) Subconfluent HPAEC were treated with 5 μM AG1024 or DMSO for 30 minutes and subjected to 50 Gy X-rays. At 6 hours post-irradiation, whole cell lysates were prepared and western blotting for cleaved, caspase-3 was performed. (B) Subcofluent cultures of HPAEC were added with 10 × IGF-1 concentration (50 ng/ml) for 18 hours and exposed to 10 Gy X-rays. At 6 hours post-irradiation, whole cell lysates were prepared and western blotting for cleaved, caspase-3 was performed.

A number of studies have shown that bypassing or blocking cellular senescence sensitizes cells to apoptosis following cellular stress [53,54]. In order to determine whether IGF-1R may play a role in this dynamics, we treated HPAEC with AG1024 (IGF-1R inhibitor) and exogenous IGF-1 (IGF-1R activator) and then exposed to X-rays. For this purpose, we used 50 Gy X-rays as we previously determined that this dose was effective in detecting caspase-3 activation in bovine PAEC [33]. Treatment with either AG1024 or exogenous IGF-1 did not alter the levels of active caspase-3 suggesting that IGF-1R does not regulate radiation-induced apoptosis (Figure 7A, B).

## Discussion

Exposure to various stresses, especially to DNA damaging agents, triggers complex cellular responses that result in either cell survival or cell death. In most cases, cellular stress may also give rise to accelerated senescence, a form of irreversible cell cycle arrest with a complex phenotype that includes extensive morphological alterations, secretion of senescence-associated proteins, and an increase in SA-β-gal activity [1]. We previously demonstrated that accelerated senescence is the primary mechanism of radiation-induced loss of clonogenicity in non-immortalized, non-cancer endothelial cells [33]. In the current study, we provide evidence that IGF-1R activation is required for IR-induced accelerated senescence. The inhibition of IGF-1R suppresses downstream activation of p53 and p21/waf1. Additionally, cells treated with the IGF-1R inhibitor maintain normal morphology and do not express SA-β-gal in response to X-ray irradiation. This is the first demonstration of the requirement of IGF-1R for accelerated senescence by ionizing radiation. 

An accumulating body of evidence suggests that cellular senescence is a state of continuous cell growth signaling in the presence of a cell cycle blockade [4]. mTOR activation was suggested to be a critical effector of the continuous cell growth component of cellular senescence. Consistent with these findings, our results indicate that radiation-induced cellular senescence involves mTOR. The inhibition of mTOR by rapamycin attenuates SA-β-gal activity induced by radiation. Interestingly, we also find that inhibition of mTOR results in attenuation of radiation-induced p21/waf1 expression, suggesting that mTOR is possibly upstream of p21/waf1 in HPAEC following radiation exposure. Indeed, a number of studies have demonstrated a crosstalk between p53 and p21/waf1 with mTOR pathways via regulations of mouse double minute 2 homolog (MDM2) and sirtuin 1 (SIRT1), both of which modulate p53 accumulation [55-57]. The contribution of each of these pathways to radiation-induced accelerated senescence in primary cells remains to be further investigated.

Our current findings in HPAEC indicate that IGF-1R activation does not inhibit radiation-induced apoptosis. Paradoxically, a number of studies have demonstrated positive effects of IGF-1R and IGF-1 in conferring radiation resistance, mostly through prevention of apoptosis in some cancer cell types [25,27,28]. However, in other tumor cell types IGF-1R is dispensable for the induction of radiation resistance, suggesting that IGF-1R is anti-apoptotic in specific cell types and that the underlying mechanism(s) of radiation resistance in other cancer cells remain unresolved [29]. Our finding of the requirement of IGF-1R for radiation-induced accelerated senescence in primary endothelial cells is consistent with the findings of a previous study on the requirement of functional IGF-1R for the initiation of UVB-induced premature senescence in primary human keratinocytes [15]. However other IGF-1R-independent signaling mechanism(s) are likely activated for radiation-induced apoptosis in primary endothelial cells. 

The generation of ROS by ionizing radiation is the primary toxic stress that causes cellular macromolecular damage [50] (Leach, Van Tuyle et al. 2001). Inhibition of the catalytic activity of phosphatases by ROS, especially protein tyrosine phosphatases, has been proposed as a mechanism for activation of kinases [58-60]. ROS were demonstrated to induce the phosphorylation of IGF-1R in transformed cells, although it has not been determined whether this activation is direct or indirect through phosphatase inactivation [52]. In agreement with these results, our data indicate that in primary HPAEC, ROS can also mediate IGF-1R activation. Our data indicate that the antioxidant NAC attenuated early IGF-1R phosphorylation induced by radiation. Together these findings imply the contribution of an ROS-dependent, ligand-independent mechanism for early activation of IGF-1R. However, our findings also indicate that a ligand-dependent mechanism contributes to delayed IGF-1R activation following radiation exposure, as revealed by the increase in both IGF-1 mRNA and secreted protein. The increase in IGF-1 levels concomitant with increased IGF-2 for IGF-1R activation may contribute to a cycle of autocrine and paracrine signaling, which in HPAEC has been demonstrated to induce cell proliferation. In the presence of the cell cycle inhibitor p21/waf1, this signaling may ultimately lead to the development of the complex senescent phenotype [1]. 

The importance of accelerated senescence in radiation-induced damage is being increasingly recognized [45]. However, the mechanisms of radiation-induced cellular senescence remain incompletely understood. Although senescent cells are metabolically active, they are incapable of replication and have altered cellular activities [61,62]. Importantly, radiation-induced cellular senescence may deplete the pool of proliferative cells thus contributing to tissue repair failure following radiation exposures [26]. The identification of specific pathways and mechanisms for radiation-induced cellular senescence may provide novel targets for the prevention of the adverse effects of clinical radiation treatments or from accidental radiation exposures. 

## Supporting Information

Figure S1 **Radiation-induced accelerated senescence is not attenuated by p42/p44 MAPK inhibition.** Subconfluent HPAEC were pretreated with 10 μM U0126 or vehicle (DMSO) for 30 minutes. Cells were either sham-irradiated (control) or exposed to 10 Gy X-rays and then incubated until time of assay. Representative pictures of SA-β-gal staining at 4 days post-irradiation are shown.
(TIF)

Table S1 **Human Cellular Senescence Array.** Subconfluent cultures of HPAEC were irradiated (10 Gy) or sham-irradiated. At 72 hours post-irradiation, 1.0 μg of RNA was subjected to reverse transcription in a total volume of 20 ml using High Capacity RNA-cDNA mix kit (Applied Biosystems) and a human cellular senescence array analysis was performed according to manufacturer’s instructions (Qiagen, SABiosciences) (n = 1). Genes with at least 1.5 fold upregulation or downregulation were tabulated.
(DOCX)
