# Correction: Inhibition of IGF-1R Prevents Ionizing Radiation-Induced Primary Endothelial Cell Senescence

**DOI:** 10.1371/annotation/70c32021-3b83-406a-9fc8-6ded27cf44f6

**Published:** 2014-01-06

**Authors:** Ronald Allan M. Panganiban, Regina M. Day

A funding organization and multiple grants to the second author (RMD) were incorrectly omitted from the Funding Statement. The Funding Statement should read:

"This study was supported by a USUHS Intramural Grant, and a USUHS predoctoral grant. The work was additionally funded by Defense Threat Reduction Agency grant H.1006_09_US_R and Defense Medical Research and Development Program grant D10_I_AR_J7_119 from the US Department of Defense to RMD. The funders had no role in study design, data collection and analysis, decision to publish, or preparation of the manuscript." 

