# Correction: Inhibition of IGF-1R Prevents Ionizing Radiation-Induced Primary Endothelial Cell Senescence

**DOI:** 10.1371/journal.pone.0096589

**Published:** 2014-04-24

**Authors:** The PLOS ONE Staff

Figure 2 is truncated in the XML and PDF versions of the article. The journal apologizes for this error, which was introduced while the manuscript was being prepared for publication by production. Please see the correct Figure 2 below.

**Figure 2:**
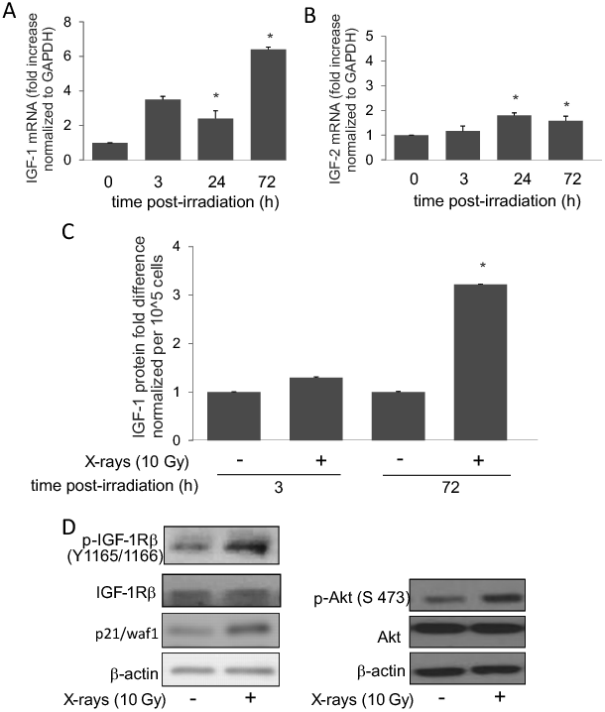
Induction of IGF-1R signaling in irradiated HPAEC. (A and B) IGF-1 and IGF-2 mRNA levels in irradiated HPAEC were assessed by qPCR at indicated time-points post-irradiation. The mRNA levels were normalized to GAPDH. Graph represents means ± SD, n  =  3. * indicates statistical significance from controls, p < 0.05. (C) IGF-1 levels in secreted medium were measured by ELISA at indicated time-points post-irrradiation. Graph represents means ± SD, n  =  3. * indicates statistical significance from controls, p < 0.05. (D) Western blotting for expressions of proteins involved in IGF-1R hyperphosphorylation (left panel) and Akt hyperphosphorylation (Ser 473, right panel) at 3 hours post-irradiation.
