# Supplementary figures and images for: Inhibition of IGF-1R Prevents Ionizing Radiation-Induced Primary Endothelial Cell Senescence
Source: PLoS One. 2013 Oct 24;8(10):e78589. doi: 10.1371/journal.pone.0078589 (PMC3813482; doi:10.1371/journal.pone.0078589)

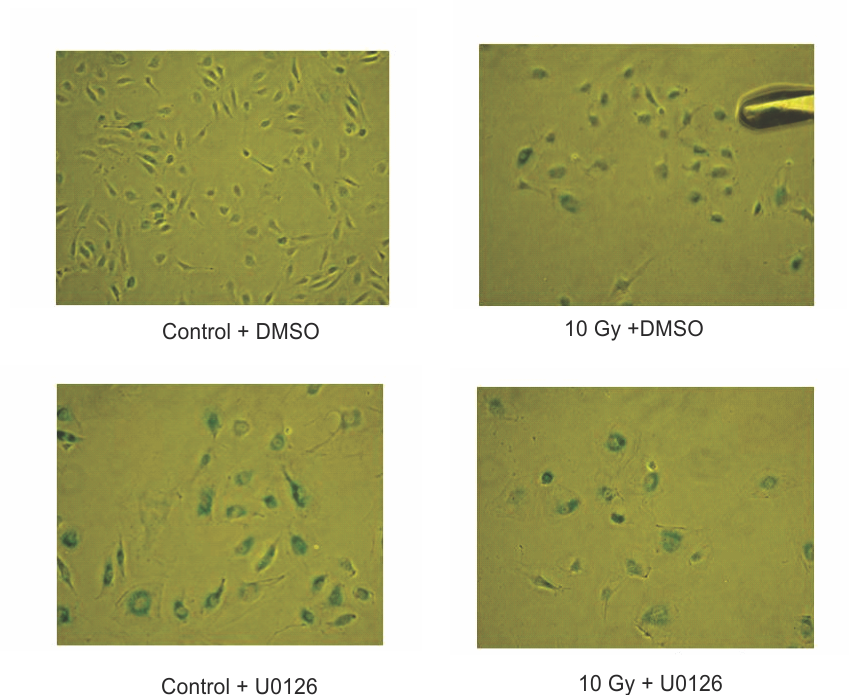

Supplement: Figure S1 — Radiation-induced accelerated senescence is not attenuated by p42/p44 MAPK inhibition. Subconfluent HPAEC were pretreated with 10 μM U0126 or vehicle (DMSO) for 30 minutes. Cells were either sham-irradiated (control) or exposed to 10 Gy X-rays and then incubated until time of assay. Representative pictures of SA-β-gal staining at 4 days post-irradiation are shown. (TIF) [file pone.0078589.s001.tif]
