# Supplementary material for: Inhibition of IGF-1R Prevents Ionizing Radiation-Induced Primary Endothelial Cell Senescence
Source: PLoS One. 2013 Oct 24;8(10):e78589. doi: 10.1371/journal.pone.0078589 (PMC3813482; doi:10.1371/journal.pone.0078589)
Supplement: Table S1 — Human Cellular Senescence Array. Subconfluent cultures of HPAEC were irradiated (10 Gy) or sham-irradiated. At 72 hours post-irradiation, 1.0 μg of RNA was subjected to reverse transcription in a total volume of 20 ml using High Capacity RNA-cDNA mix kit (Applied Biosystems) and a human cellular senescence array analysis was performed according to manufacturer’s instructions (Qiagen, SABiosciences) (n = 1). Genes with at least 1.5 fold upregulation or downregulation were tabulated. (DOCX) [file pone.0078589.s002.docx]

**Table 1. Downregulated and upregulated genes post-irradiation as identified by human cellular senescence array.**

| **DOWNREGULATED** |  |  |
| --- | --- | --- |
| **Gene Symbol** | **Description** | **Fold** |
| ALDH1A3 | Aldehyde dehydrogenase 1 family, member A3 | -2.46 |
| ATM | Ataxia telangiectasia mutated | -2.29 |
| BMI1 | BMI1 polycomb ring finger oncogene | -1.83 |
| CCNA2 | Cyclin A2 | -25.25 |
| CCNB1 | Cyclin B1 | -12.54 |
| CCNE1 | Cyclin E1 | -1.55 |
| CDC25C | Cell division cycle 25 homolog C (S. pombe) | -98.22 |
| CDK2 | Cyclin-dependent kinase 2 | -2.55 |
| CDK4 | Cyclin-dependent kinase 4 | -1.89 |
| CDKN1B | Cyclin-dependent kinase inhibitor 1B (p27, Kip1) | -3.53 |
| CDKN1C | Cyclin-dependent kinase inhibitor 1C (p57, Kip2) | -6.01 |
| CDKN2A | Cyclin-dependent kinase inhibitor 2A (melanoma, p16, inhibits CDK4) | -1.62 |
| CDKN2C | Cyclin-dependent kinase inhibitor 2C (p18, inhibits CDK4) | -12.54 |
| CHEK1 | CHK1 checkpoint homolog (S. pombe) | -2.8 |
| CHEK2 | CHK2 checkpoint homolog (S. pombe) | -2.94 |
| COL3A1 | Collagen, type III, alpha 1 | -2.26 |
| E2F1 | E2F transcription factor 1 | -17.98 |
| E2F3 | E2F transcription factor 3 | -1.74 |
| EGR1 | Early growth response 1 | -2.1 |
| ETS1 | V-ets erythroblastosis virus E26 oncogene homolog 1 (avian) | -3.22 |
| ETS2 | V-Ets erythroblastosis virus E26 oncogene homolog 2 (avian) | -1.64 |
| ING1 | Inhibitor of growth family, member 1 | -2.16 |
| MAP2K1 | Mitogen-activated protein kinase kinase 1 | -1.68 |
| MAPK14 | Mitogen-activated protein kinase 14 | -2.55 |
| MORC3 | MORC family CW-type zinc finger 3 | -1.68 |
| MYC | V-myc myelocytomatosis viral oncogene homolog (avian) | -1.9 |
| NFKB1 | Nuclear factor of kappa light polypeptide gene enhancer in B-cells 1 | -1.63 |
| PCNA | Proliferating cell nuclear antigen | -1.89 |
| PIK3CA | Phosphoinositide-3-kinase, catalytic, alpha polypeptide | -1.92 |
| PTEN | Phosphatase and tensin homolog | -1.86 |
| RB1 | Retinoblastoma 1 | -1.67 |
| RBL1 | Retinoblastoma-like 1 (p107) | -4.34 |
| RBL2 | Retinoblastoma-like 2 (p130) | -1.97 |
| SIRT1 | Sirtuin (silent mating type information regulation 2 homolog) 1 (S. cerevisiae) | -2.04 |
| SOD2 | Superoxide dismutase 2, mitochondrial | -1.76 |
| TBX2 | T-box 2 | -2.2 |
| TBX3 | T-box 3 | -1.73 |
| TERF2 | Telomeric repeat binding factor 2 | -2.13 |
| TERT | Telomerase reverse transcriptase | -2.86 |
| TGFB1 | Transforming growth factor, beta 1 | -1.7 |
| TGFB1I1 | Transforming growth factor beta 1 induced transcript 1 | -1.64 |
| TP53BP1 | Tumor protein p53 binding protein 1 | -1.64 |
| TWIST1 | Twist homolog 1 (Drosophila) | -3.05 |
|  |  |  |
|  |  |  |
| **UPREGULATED** |  |  |
| **Gene Symbol** | **Description** | **Fold** |
| CCND1 | Cyclin D1 | 1.71 |
| CD44 | CD44 molecule (Indian blood group) | 2.21 |
| CDKN1A | Cyclin-dependent kinase inhibitor 1A (p21, Cip1) | 6.69 |
| CDKN2B | Cyclin-dependent kinase inhibitor 2B (p15, inhibits CDK4) | 2.22 |
| FN1 | Fibronectin 1 | 1.6 |
| IGF1 | Insulin-like growth factor 1 (somatomedin C) | 14.14 |
| IRF7 | Interferon regulatory factor 7 | 2.77 |
| MDM2 | Mdm2 p53 binding protein homolog (mouse) | 2.85 |
| NBN | Nibrin | 534.48 |
| PLAU | Plasminogen activator, urokinase | 2.41 |
| SERPINB2 | Serpin peptidase inhibitor, clade B (ovalbumin), member 2 | 18.92 |
| SERPINE1 | Serpin peptidase inhibitor, clade E (nexin, plasminogen activator inhibitor type 1), member 1 | 1.98 |

Subconfluent cultures of HPAEC were irradiated (10 Gy) or sham-irradiated. At 72 hours post-irradiation, total RNA was harvested, reverse-transcribed, and subjected to human cellular senescence array analysis (n=1). Genes with at least 1.5 fold upregulation or downregulation were tabulated.
